# C-954-14. Resource Support to Optimize Recovery: A Qualitative Study to Examine Perspectives on Burn Aftercare Resources

**DOI:** 10.1093/jbcr/irag033.146

**Published:** 2026-04-06

**Authors:** Renee C Noordzij, Carla Tierney-Hendricks, Diana L Tenney-Laperriere, Amy L Acton, Jill L Sproul, Huan Deng, Lewis E Kazis, Colleen M Ryan, Mary D Slavin, Jeffrey C Schneider

**Affiliations:** Boston University, Boston, MA; Spaulding Rehabilitation Hospital, Harvard Medical School, Boston, MA; Boston University School of Public Health, Boston MA; Phoenix Society for Burn Survivors, Kentwood, MI; Phoenix Society for Burn Survivors, San Jose, CA; Spaulding Rehabilitation Hospital, Harvard Medical School, Boston, MA; Boston University School of Public Health, Boston MA; Massachusetts General Hospital, Harvard Medical School, Boston, MA; Massachusetts General Hospital Brigham - Spaulding, Boston, MA; Spaulding Rehabilitation Hospital, Mass General Brigham, Harvard Medical School, Boston, MA

## Abstract

**Introduction:**

The aftercare phase of recovery, involving the transition from acute hospital care or inpatient rehabilitation to living in the community, can be challenging for burn survivors. Burn centers across the U.S. lack standardized guidelines for providing quality burn injury aftercare resources. This qualitative study engaged burn survivors, caregivers, and burn care providers to better understand priorities and preferences for providing aftercare resources.

**Methods:**

Focus groups and semi-structured interviews were conducted, inquiring about resource content, delivery and implementation processes. The World Health Organization International Classification of Functioning, Health, and Disability provided the conceptual framework for deductive coding. The codebook was iteratively reviewed and revised. Two members of the research team used the final codebook to independently code transcripts using thematic analysis. A third team member reviewed the coding and resolved discrepancies.

**Results:**

The sample included 42 participants: burn survivors (n = 17); caregivers (n = 2); and burn care providers (n = 23). Participants were mostly female (76%), white (81%), and not Hispanic (88%). Providers represented various professional roles within burn centers. ICF Themes: Body Function sub-themes: wound care, scar management, and therapy needs. Activity & Participation sub-themes: peer relations and mental health. Personal Factors sub-themes: every burn survivor is different, relationships in recovery, and access to aftercare resources. Environmental Factors sub-themes: navigating health insurance and social security and the general public and stigma. Delivery Themes: connecting burn survivors to resources by laying out the recovery map and preference for multi-modal formats and in-person discussion. Implementation Themes: lack of funding and standardization of aftercare as barriers and training for burn care providers as a potential facilitator.

**Conclusions:**

Study findings identify priorities for burn injury aftercare resource content and suggest strategies to enhance resource delivery and promote implementation.

**Applicability of Research to Practice:**

This study will inform a modified Delphi process focused on standardizing the content of aftercare resources and recommendations for delivery and implementation processes.

**Funding for the study:**

This work is supported by the National Institute on Disability, Independent Living, and Rehabilitation Research (#90DPBU0008).

